# Evaluation of the Effects of Processing Technique on Chemical Components in Raphani Semen by HPLC and UPLC-Q-TOF-MS

**DOI:** 10.1155/2022/8279839

**Published:** 2022-01-04

**Authors:** Sijia Gao, Jirui Wang, Lei Cheng, Yuxin Fan, Weihan Qin, Yunhong Wang, Yanlei Guo, Xiaomei Zhang, Yong Yang

**Affiliations:** ^1^Chongqing Academy of Chinese Materia Medica, Chongqing, China; ^2^College of Pharmacy, Chengdu University of Traditional Chinese Medicine, Chengdu, Sichuan, China

## Abstract

In this study, the effects of different processing techniques on the chemical components of Raphani Semen (RS) were evaluated. An established high-performance liquid chromatography (HPLC) method was adopted for the simultaneous determination of glucoraphanin, sinapine thiocyanate, raphanin, and erucic acid in the fried products of Raphani Semen to evaluate the chemical changes during frying processing as well as optimize the best frying technology of Raphani Semen. Then, the chemical components in the fried Raphani Semen were identified by ultrahigh-performance liquid chromatography-quadrupole time-of-flight mass spectrometry (UPLC-Q-TOF-MS). A total of 54 compounds in processed Raphani Semen were identified by UPLC-Q-TOF-MS. The results showed that the content of glucoraphanin and sinapine thiocyanate was the highest in the fried products at 130°C for 10 min, and the effect of “Enzyme Killing and Glycosides Preserving” was the best. Therefore, this condition was chosen as the best frying technology of Raphani Semen. This study provided a more scientific basis for evaluation of the quality of Raphani Semen fried products and optimization of the frying technology of Raphani Semen.

## 1. Introduction

Processing, pronounced as Paozhi in Chinese, is recognized as an ancient Chinese pharmaceutic technique developed over thousands of years to increase efficiency and decrease toxicity of herbs in the guidance of traditional Chinese medicine (TCM) theory [1]. According to TCM theory, processing can alter energetic nature and therapeutic direction, as well as improve flavor of herbal medicines, thereby increasing the therapeutic effectiveness and applicability in clinics. Most Chinese herbal medicines need to be properly processed to become decoction pieces and obtain the specific drug efficiency [2]. Processing technique encompasses roasting, baking, and stir-frying with or without liquid/solid excipient, by which decoction pieces with different therapeutic potencies can be derived from the same herb [3]. For instance, Coptidis rhizome (CR) is a commonly used Chinese medicine in clinics for the treatment of various inflammatory disorders and related diseases with the functions of clearing heat, drying dampness, and detoxification [4–6]. There were four processed CR, namely, raw CR, wine CR (stir-frying with wine), ginger CR (stir-frying with ginger), Evodiae Fructus CR (stir-baking with Evodiae Fructus) in the Chinese Pharmacopoeia (2020 Edition). Different processing methods are developed to guide and concentrate therapeutic effects of CR. Wine CR is inclined to treat insomnia, sore mouth, and red and swelling eyes [7–9], and ginger CR is prescribed for enhancing the effect preventing vomiting and expelling phlegm [10], while Evodiae Fructus CR is mainly used for curing diarrhea [7]. In a nutshell, processing is essential for safety and effectiveness of TCM as it may cause complicated changes in active chemical components, such as the contents may alter, the structures may change, and new compound occurred, and therefore, the functions of herbal medicines. But the underlying mechanisms of processing remain unclear for most TCM; therefore, investigation of the chemical changes of TCM before and after processing is key for understanding of underlying mechanisms.

Raphani Semen (RS), the dried and mature seed of *Raphanus sativus* L., was first recorded in the Chinese herbal medicine “RiHuaZi Materia Medica” in the Five Dynasties [11]. It is pungent and sweet in flavor and has the effect of eliminating food and distension, qi-descending, and phlegm-resolving [12]. Therefore, it has been used for stagnation of diet, abdominal distension, constipation, retention of diarrhea, phlegm, and asthma in clinics. The chemical components of RS mainly include glucosides, alkaloids, volatile oils, fatty acids, flavonoids, and polysaccharides [13]. Pharmacological studies showed that RS had the functions of relieving asthma, suppressing cough, removing phlegm, antioxidation, and enhancing gastrointestinal motility [14, 15]. Processing has been a major feature of TCM, and RS is one of the typical examples. In the theory of TCM, processing would affect the efficacy of RS, the unprocessed RS is good at inducing sputum, and after the frying process, it is good at reducing qi, resolving phlegm and eliminating food [16]. At present, fried RS is more widely used in clinics, and research studies have evaluated the contents change of glucoraphanin and sinapine thiocyanate before and after the frying process. Previous studies in China showed that the frying of RS played a role of “Enzyme Killing and Glycosides Preserving,” inhibiting the activity of mustard enzyme in RS and preventing the decomposition of glucoraphanin [17]. It was indicated that the sulfur compounds were one of the material bases of RS with the characteristics of the efficacy varied with processing, which can be used as a specific quality control index to effectively reflect the degree of frying RS at the same time.

However, there were no reports on the identification of the active components and the changes of the components under different frying conditions. Through investigation, referring to the actual production of pharmaceutical enterprises and the pretest of the experiment in the early stage of our research group, the chemical components of the fried products of RS were qualitatively identified under different firing conditions. By determining the content of glucoraphanin, raphanin, erucic acid, and sinapine thiocyanate in RS, taking the content of water-soluble glucoraphanin as the main measurement standard, combined with the content of other three components and the overall profiles as auxiliary indexes, the frying process of RS was discussed, hoping to provide reference for the frying process and clinical application of RS.

## 2. Materials and Methods

### 2.1. Medicinal Materials

The RS used in the current experiment was purchased in the year of 2020 from Chongqing Shangyao Huiyuan Pharmaceutical Co., Ltd. and identified as the dried and mature seed of *Raphanus sativus* L. by Prof. Xianyou Qu, researcher of Institute of Pharmacognosy, Chongqing Academy of Chinese Materia Medica.

### 2.2. Chemicals, Reagents, and Instrumentation

The reference compounds sinapine thiocyanate (purity ≥98.0%, batch number PS011003) and glucoraphanin (purity ≥ 95.0%, batch number PS011849) were purchased from Chengdu Pusi Biotechnology Co., Ltd. The reference compounds raphanin (purity ≥ 98.0%, batch number BP1814) and erucic acid (purity ≥ 98.0%, batch number BP1778) were purchased from Chengdu Pulifa Technology Development Co., Ltd. Water used for all analyses was ultrapure water, the acetonitrile and methanol were chromatographic pure, and other reagents were analytical pure.

A Waters 2695 HPLC (Waters, Milford, MA, USA), Shimadzu LC-30A UPLC, and Triple TOF™ 600 Q-TOF-MS (Allen-Bradley company, USA, including Analyst 1.6 workstation, PeakView 1.2.0.3 data processing software) were adopted to conduct the content determination and quantitative analysis of RS. Shimadzu AEG-45SM five-decimal electronic balance (Shimadzu, Kyoto, Japan) and BP121S One Over Ten Thousand Analytical Balance (Sartorius company, Germany) were used to weight. CY-25 Chinese medicine frying machine (Wenzhou Dingli Medical Instrument Co., Ltd.) was used to process RS.

### 2.3. Preparation of Fried Products of RS

Referring to the processing technology of RS from two pharmaceutical companies in Chongqing and the preliminary experiment of our research group, the processing conditions were set as given in Table 1. About 500 g of RS was put in the frying machine, and the processing temperature and time for frying were set as given in Table 1.

### 2.4. Standard Preparation and Calibration Curve

The reference compounds glucoraphanin, sinapine thiocyanate, raphanin, and erucic acid were accurately weighed and dissolved in 1 mL 50% methanol to produce a mixed standard stock solution with the concentrations of 0.256 mg/mL, 0.0521 mg/mL, 0.193 mg/mL, and 0.0357 mg/mL, respectively. And 0.5, 1, 2, 5, 10, 20, and 30 *μ*L of the standard mixture were separately taken and diluted in 50% methanol for HPLC analysis to make calibration curves.

### 2.5. Sample Preparation

Half gram of fried RS powder was extracted by ultrasonic (power 250 W, frequency 40 Hz, temperature 30°C) with 50 mL water for 30 min and filtered.

### 2.6. Chromatographic Conditions

HPLC condition: chromatographic column: Agilent SB-C_18_ (4.6 × 250 mm, 5 *μ*m); mobile phase: acetonitrile (A)–0.1% phosphoric acid solution (B), gradient elution (0∼15 min, 5% A ⟶ 8% A; 15–25 min, 8%A ⟶ 20% A; 25–40 min, 20% A ⟶ 24% A; 40–50 min, 24% A ⟶ 45% A; 50–52 min, 45% A ⟶ 5% A; 52–55 min, 5% A ⟶ 5% A); detection wavelength: 225 nm; column temperature: 30°C; current velocity: 1 mL∙min^−1^; sample size: 10 *μ*L. The compounds were quantified by dividing the peak areas of the compounds of interest by the peak area of the standard compound [18].

UPLC-Q-TOF-MS condition: the chromatographic column adopted in current study was ACE Excel Super C_18_ chromatographic column (2.1 × 100 mm, 3 *μ*m), and column temperature was 30°C. The mobile phase was acetonitrile (A)–0.1% formic acid aqueous solution (B) with gradient elution (0-1 min, 15% A; 1–8.5 min, 15%A ⟶ 85%A, 8.5–11.5 min, 85%A; 11.5-11.6 min, 85%A ⟶ 15%A; 11.6–15 min, 15%A). The flow rate was set at 0.25 mL/min, and the injection volume was 3 *μ*L.

Mass spectrometry condition: ESI-positive IDA mode was used to collect data. ISVF: +5.5 kV; GS1: 0.40 MPa; CUR: 0.14 MPa; GS2: 0.35 MPa; TEMP: 600°C; DP: 60 V; CE: 50 V; CES: ±15 V. The detection mode was IDA, MMDF and DBS were the conditions to trigger the second level scanning, and the second level scanning was preferred when the conditions were met.

### 2.7. Data Analysis

Referring to the related literature on the chemical constituents of RS, a high-resolution mass spectrometry screening database for chemical components of RS was constructed. PeakView software was used to extract and analyze the 16 batch samples data of different processing conditions collected by Q-TOF, and the target compounds were the ions with mass error less than 5 ppm, correct isotope distribution, and secondary fragments. Combined with the functions of formula finder, mass calculators, online databases (ChemSpider, METLIN, and HMDB) and secondary fragmentation rules, the chemical structures of the known components of RS were identified and verified.

## 3. Results

### 3.1. Method Development and Validation

**Figure 1:**
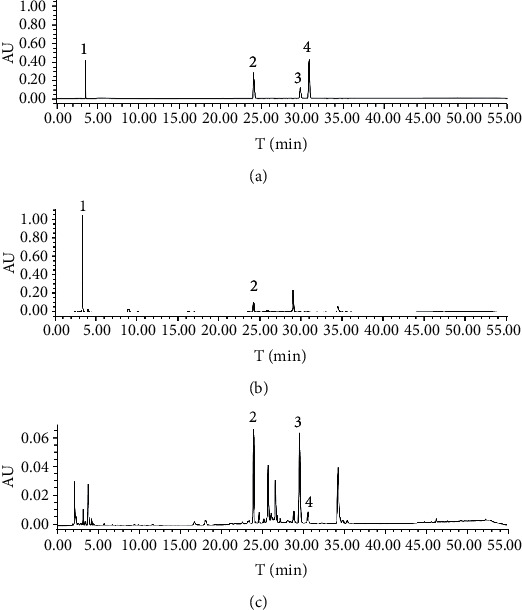
HPLC chromatograms of mixed reference substances. (a) *Raphanus semen* in 240°C for 10 min (b) and 120°C for 20 min (c). 1, glucoraphanin; 2, sinapine thiocyanate; 3, raphanin; 4, erucic acid.

Detailed experimental results can be found in the previous study published by our research group [19]. As shown in Figure 1, the HPLC method ensured sufficient chromatographic separation and accurate and precise quantification of glucoraphanin, sinapine thiocyanate, raphanin, and erucic acid. The linear regression equations obtained for four analytes are given in Table 2, and the sample concentrations in this experiment were within the established linear range. The HPLC method was validated for its linearity, precision, stability, repeatability, and recovery. The results of instrumental precision showed that the RSDs% of peak areas for four analytes (glucoraphanin, sinapine thiocyanate, raphanin, and erucic acid) were less than 1% (0.97%, 0.78%, 0.81%, and 0.97%, respectively), indicating that the analysis instrument was in good precision condition. The RSDs% (in the reference compounds) results of glucoraphanin, sinapine thiocyanate, raphanin, and erucic acid were 0.86%, 0.89%, 0.46%, and 0.79%, respectively; The RSDs% (in the samples) results of glucoraphanin, sinapine thiocyanate, raphanin, and erucic acid were 1.04%, 0.78%, 0.91%, and 1.27%, respectively, which indicated that the stability of reference solution and sample solution was good within 48 h. All samples in this experiment were tested within 48 h from sample preparation. The repeatability assessment in the established method shown that RSDs% of glucoraphanin, sinapine thiocyanate, raphanin, and erucic acid were 1.01%, 1.26%, 1.08%, and 1.32%, respectively, which indicated that the method was with good repeatability. The established method also had acceptable accuracy with spike recovery, which were 100.14%, 99.61%, 99.05%, and 99.52%, respectively, with RSDs% of 1.94%, 2.34%, 1.86%, and 2.17% for glucoraphanin, sinapine thiocyanate, raphanin, and erucic acid, respectively. The method was successfully employed for quantitative determination of glucoraphanin, sinapine thiocyanate, raphanin, and erucic acid in raw and fried RS samples.

### 3.2. Effects of Processing Technique on RS

As given in Table 3, the glucoraphanin was not detected in the water extract of RS after frying at 70–90°C for 10–40 min. With the increase of frying time to 110°C, the content of glucoraphanin increased gradually. At temperature of 130°C, the content of glucoraphanin decreased gradually to undetectable with the increased frying time in the range of 10–40 min. Samples frying at 130°C for 10 min obtained the highest content of glucoraphanin. The results indicated that the content of glucoraphanin is closely related to the processing time and temperature. At a lower processing temperature, glucoraphanin will be hydrolyzed by myrosinase during the extraction process with water as the medium. When the processing temperature is too high, glucoraphanin may be directly destroyed. For sinapine thiocyanate, when the temperature is from 70°C to 110°C, with the increased frying temperature and time, the content of it in water extract also increased. The content of sinapine thiocyanate was the highest when frying at 130°C for 10 min, and with the increase of frying time, the content of which decreased rapidly. When it comes to raphanin, at the frying temperature of 70–90°C, the content of raphanin increased gradually. Reaching the temperature of 110°C, with the increased frying time, the content of raphanin decreased rapidly to undetectable. The results showed that raphanin and glucoraphanin hardly coexist in the same water-extracted sample. By literature review, it was found that the glucoraphanin was hydrolyzed to raphanin by myrosinase, which exactly explained the phenomenon of the current experiment [20, 21]. The content of erucic acid was not affected significantly by frying time at the temperature of 70°C. But when the frying temperature reached 90°C, content of erucic acid began to decrease to undetectable with the increase of temperature and time.

### 3.3. Comparison of the Overall Profile

**Figure 2:**
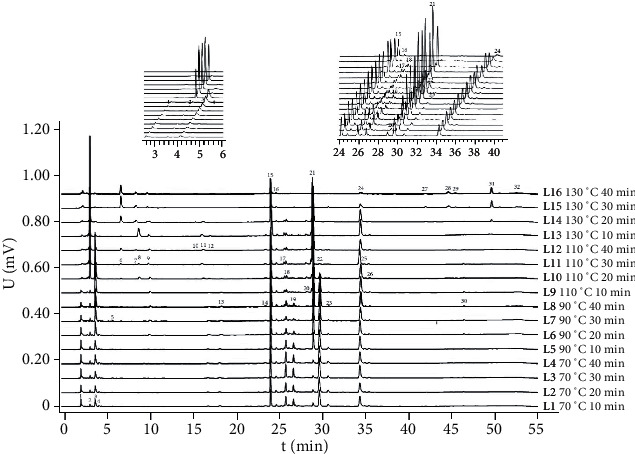
Overall chromatograms of 16 batches of RS-processed products.

In order to comprehensively compare the changes of components in RS during the frying process, the overall profiles of chemical components in RS at different frying temperature and time were overlapped [22]. The results are shown in Figure 2. From the overall profiles of different fried products, 32 main chromatographic peaks were detected in 16 samples. With the increased frying temperature and frying time, the number and height of chromatographic peaks before 10 min were increased, indicating that high frying temperature could preserve the large polar components including glucoraphanin (peak 2). The peaks in 24–30 min were also affected by the frying process. The peak area of No. 15 peak (sinapine thiocyanate) changed more smoothly and showed an overall upward trend, but long-time frying at high temperature could also destroy the component, which was consistent with the determination of chemical composition. The peak areas of No. 18, No. 19, No. 22 (raphanin), and No. 23 (erucic acid) decreased with the increase of frying temperature and time, while the peak areas of No. 21 showed an increasing trend. The peak area of No. 24 also decreased sharply when fried for a long time at high temperature (L15 and L16), while the peak area of No. 27, No. 28, No. 29, No. 31, and No. 32 gradually increased under high temperature (L14, L15, and L16).

### 3.4. Identification of the Chemical Composition of Processed RS

**Figure 3:**
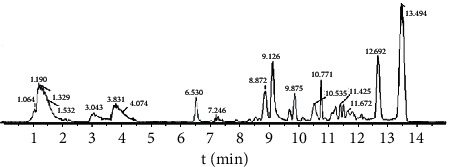
Total ion chromatogram of processed RS (*Raphanus semen* frying in 240°C for 10 min).

In this study, the UPLC-Q-TOF-MS method was further introduced to analyze and identify chemical components of RS in combination with online database. The total ion chromatogram of processed RS is shown in Figure 3. The molecular formula information of the compound was obtained by PeakView1.2 to fit the element composition from the first-order MS. The structures of each compound were inferred by analyzing the fragmentation law of MS combined with the network databases such as HMDB and MassBank. A total of 54 chemical components in fried RS were identified in this study, including thioglycoside, alkaloids, volatile oils, fatty acids, and other components, as given in Table 4.

## 4. Discussion

In general, enzyme (myrosinase) hydrolysis occurs in chemical components of raw RS during rinsing or warm water treatment, and to prevent the active ingredients from enzyme hydrolysis, the processing technique was introduced to medicinal herbs in TCM clinics. Usually, through heating and frying, the activity of myrosinase can be inhibited [23], so as to achieve the effect of “Enzyme Killing and Glycosides Preserving,” which reflects the characteristics of the efficacy varied with processing [24]. The myrosinase in raw RS can play its enzymatic activity in water, transfer hydrolyze glucoraphanin to raphanin, and stir frying can make the myrosinase lose its activity, while using water heating extraction will not lead to the decomposition of glucoraphanin, so using water as the extraction solvent can reflect whether the frying degree is appropriate; thus, water was used as the extraction solvent in this study. In the current study, we found that different frying temperature and time had great influence on the chemical composition of RS. A small amount of glucoraphanin could be detected in samples frying at 110°C for 20 min, which indicated that myrosinase was inhibited, but not completely inactivated. The content of glucoraphanin reached the maximum at 130°C for 10 min, which indicated that this condition had the strongest inhibitory effect on myrosinase. However, with the increase of frying time, the content of glucoraphanin began to decline or even could not be detected. At the same time, the samples also began to change from the fried state to the scorched state, indicating that if the frying time was too long, glucoraphanin would be destroyed and decomposed by high temperature. When fried at 130°C for 10 min, the contents of glucoraphanin and sinapine thiocyanate in the fried products were the highest, and the chromatographic peaks of many unknown components in the overall profile were also higher. Under this condition, the fried products of RS were bulging, and the seed coat was easy to twist off, with rich in oil and special aroma and high identification of external properties. Therefore, according to the changes of chemical composition and the characteristics, the frying process of RS at 130°C for 10 min was selected as the best processing condition. This study provided the basis for scientific evaluation of the quality control of RS and standardization of the frying process.

## 5. Conclusion

Our study revealed that in the process of frying RS, on the one hand, it should be prevented that the frying temperature and time were not enough to achieve the purpose of “Enzyme Killing and Glycosides Preserving,” and on the other hand, it should also pay attention to the improper frying temperature and time, which will cause scorch and destroy the effective ingredients. A total of 54 chemical components in fried RS were identified in this study, including thioglycoside, alkaloids, volatile oils, fatty acids and other components. This study only discussed the frying process of RS from the chemical composition changes, and the next step is to verify the frying process combined with pharmacodynamic research, so as to provide a more comprehensive reference for the frying process and clinical application of RS.

## Figures and Tables

**Table 1:** Sample information of RS in different processing temperature and time.

No.	*T* (°C)	*t* (min)
L1	70	10
L2	70	20
L3	70	30
L4	70	40
L5	90	10
L6	90	20
L7	90	30
L8	90	40
L9	110	10
L10	110	20
L11	110	30
L12	110	40
L13	130	10
L14	130	20
L15	130	30
L16	130	40

**Table 2:** Regression equation and linear range.

Compound	Regression equation	Linear range (*μ*g)	*r*
Glucoraphanin	*Y* = 992029.90 − 5949.49*X*	0.1280–7.680	0.9999
Sinapine thiocyanate	*Y* = 2694619.00 − 578.74*X*	0.0261–1.563	0.9999
Raphanin	*Y* = 645660.40 + 2 759.24*X*	0.0965–5.790	0.999 9
Erucic acid	*Y* = 3668510.67 + 3213.65 *X*	0.0179–1.071	0.9999

**Table 3:** The mean contents of the 4 components in RS samples by different processing technologies (*n* = 2).

No.	Glucoraphanin (%)	Sinapine thiocyanate (%)	Raphanin (%)	Erucic acid (%)
L1	—	0.25	1.09	0.02
L2	—	0.24	1.01	0.02
L3	—	0.33	1.34	0.03
L4	—	0.28	1.16	0.02
L5	—	0.32	1.30	0.02
L6	—	0.35	1.41	0.01
L7	—	0.33	1.40	0.01
L8	—	0.38	1.56	0.01
L9	—	0.38	1.35	0.01
L10	1.61	0.38	0.35	—
L11	2.84	0.39	—	—
L12	3.44	0.35	—	—
L13	3.92	0.41	—	—
L14	2.09	0.39	—	—
L15	0.07	0.30	—	—
L16	—	0.21	—	—

*Note.* “—” means that it is not detected at the current concentration.

**Table 4:** Identification of the chemical composition of processed RS.

Retention time (min)	Mass (*m/z*)		Deviation (ppm)	Formula	Components name
	Theoretical value	Measured value			
7.01	94.9984	94.9981	3.1	C_2_H_6_S_2_	Dimethyl disulfide
13.74	126.9704	126.9699	3.9	C_2_H_6_S_3_	Dimethyl trithioether
14.67	127.0060	127.0063	2.3	C_2_H_6_O_4_S	Dimethyl sulfate
14.64	158.9425	158.9421	2.5	C_2_H_6_S_4_	Dimethyl tetrasulfide
1.25	123.0297	123.0291	4.8	C_4_H_10_S_2_	1,1-Bis(methylthio)methane
3.91	113.0233	113.0232	0.8	C_5_H_4_O_3_	5-Hydroxymethylfurfural
1.56	141.0182	141.0185	2.1	C_6_H_4_O_4_	Coumalic acid
1.62	127.0390	127.0390	0.1	C_6_H_6_O_3_	5-Hydroxymethylfurfural
3.91	177.0394	177.0392	1.1	C_6_H_8_O_6_	Vitamin C
7.81	99.0804	99.0805	0.7	C_6_H_10_O	*α*-*β*-Vinyl aldehyde
4.73	119.1067	119.1066	0.8	C_6_H_14_O_2_	1,1-Dimethoxy-2-methylpropane
1.56	144.0478	144.0481	2.0	C_6_H_9_NOS	(4E)-5-(Methylsulfinyl)pent-4-enenitrile
3.90	178.0355	178.0358	1.6	C_6_H_11_NOS_2_	Glucoraphanin
3.91	176.0198	176.0204	3.3	C_6_H_9_NOS_2_	Raphanin
1.47	144.0842	144.0840	1.3	C_7_H_13_NS	Hexyl isothiocyanate
1.14	191.0736	191.0732	2.1	C_8_H_14_O_3_S	(4E)-Ethyl 5-(methylsulfinyl)pent-4-enoate,5
13.49	113.1325	113.1324	0.9	C_8_H_16_	Cyclohexane,1,3-dimethyl
2.40	181.0495	181.0499	2.2	C_9_H_8_O_4_	Caffeic acid
1.18	165.0546	165.0547	0.7	C_9_H_8_O_3_	Phenylpyruvic acid
5.95	162.0550	162.0553	2.1	C_9_H_7_NO_2_	4-Hydroxy-3-indolaldehyde
3.69	195.0652	195.0660	4.4	C_10_H_10_O_4_	Ferulic acid
1.83	209.0808	209.0805	1.4	C_11_H_12_O_4_	*trans*-Ferulic acid methyl
3.68	225.0758	225.0767	4	C_11_H_12_O_5_	Sinapic acid
10.20	223.0965	223.0971	2.6	C_12_H_14_O_4_	Ethyl ferulate
4.04	239.0914	239.0907	2.9	C_12_H_14_O_5_	Antithiamine factor
1.45	438.0557	438.0570	2.9	C_12_H_23_NO_10_S_3_	Raphthioglucoside
1.21	278.1196	278.1197	0.4	C_12_H_17_CN_4_OS	Vitamin B1
13.49	279.1591	279.1592	0.5	C_16_H_22_O_4_	Dibutyl phthalate
14.18	257.2475	257.2487	4.6	C_16_H_32_O_2_	Palmitic acid
6.86	247.1931	247.1933	0.8	C_16_H_24_NO	Sinapine
1.84	333.0969	333.0967	0.6	C_17_H_16_O_7_	Erucic acid-5-hydroxymethyl furfural ester
1.63	377.1456	377.1466	2.6	C_17_H_20_N_4_O_6_	Vitamin B2
3.20	369.1479	369.1489	2.7	C_17_H_24_N_2_O_5_S	*trans*-sinapine thiocyanate
10.41	281.2475	281.2486	3.7	C_18_H_32_O_2_	Linoleic acid
9.79	285.2788	285.2781	2.4	C_18_H_36_O_2_	Stearic acid
8.44	279.2319	279.2328	3.5	C_18_H_30_O_2_	Linolenic acid
12.26	283.2632	283.2638	2.1	C_18_H_34_O_2_	Oleic acid
10.92	311.2945	311.2943	0.6	C_20_H_38_O_2_	Eicosenoic acid
1.18	313.3101	313.3108	2.2	C_20_H_40_O_2_	Arachidic acid
1.48	452.1374	452.1358	3.5	C_21_H_25_NO_8_S	(4E)-5-[(6-O-Feruloyl)-*β*-D-glucopyranosylsulfanyl]pent-4-enenitrile
12.03	339.3258	338.3255	0.9	C_22_H_42_O_2_	Erucic acid
12.03	338.3417	338.3423	1.6	C_22_H_43_NO	Dodecanoic acid (cis-13)
1.68	479.1184	479.1207	4.7	C_22_H_22_O_12_	Isorhamnetin-3-O-glucoside
1.09	482.1479	482.1470	1.8	C_22_H_27_NO_9_S	(4E)-5-(6-O-Sinapoly)-*β*-D-glucopyranosylsulfanyl]pent-4-enenitrile
9.98	367.3571	367.3579	2.2	C_24_H_46_O_2_	Ethyl erucate
2.11	579.1708	579.1721	2.2	C_27_H_30_O_14_	Kaempferol 3,7-O-*α*-L-rhamnoside
9.52	399.3621	399.3611	2.5	C_28_H_46_O	(22E,24R)-Ergostere-5, 22-diene-3*β*-alcohol
1.71	641.1712	641.1716	0.6	C_28_H_32_O_17_	Isorhamnetin 3,4′-o-*β*-d-disglucoside
8.28	415.3934	415.3933	0.2	C_29_H_50_O	*β*-Sitosterol
9.30	412.3700	412.3694	1.4	C_29_H_47_O	Stigmast-4-en-3-one
9.44	423.4924	423.4913	2.6	C_30_H_62_	Triacontane
3.21	755.2393	755.2413	2.7	C_34_H_42_O_19_	*β*-D-(3-Sinapoyl)frucofuranosyl-*α*-D-(6-sinapoyl)glucopyranoside
11.20	577.4463	577.4450	2.2	C_35_H_60_O_6_	*β*-Stigmaster-3-O-*β*-D-glucoside
11.09	961.2972	961.2930	4.4	C_45_H_52_O_23_	*β*-D-(3,4-Disinapoyl)frucofuranosyl-*α*-D-(6-sinapoyl) glucopyranosidec

## Data Availability

The data used to support the findings of this study are available from the corresponding author upon request.

## References

[1] Zhao Z., Liang Z., Chan K. (2010). A unique issue in the standardization of Chinese materia medica: processing. *Planta Medica*.

[2] Wang S., Wu X., Tan M. (2012). Fighting fire with fire: poisonous Chinese herbal medicine for cancer therapy. *Journal of Ethnopharmacology*.

[3] Sheridan H., Kopp B., Krenn L., Guo D., Sendker J. (2015). Traditional Chinese herbal medicine preparation: invoking the butterfly effect. *Science*.

[4] Wang J., Wang L., Lou G.-H. (2019). Coptidis Rhizoma: a comprehensive review of its traditional uses, botany, phytochemistry, pharmacology and toxicology. *Pharmaceutical Biology*.

[5] Ma B.-L., Ma Y.-M. (2013). Pharmacokinetic properties, potential herb-drug interactions and acute toxicity of oral Rhizoma coptidisalkaloids. *Expert Opinion on Drug Metabolism and Toxicology*.

[6] Wang Z., Yang Y., Liu M. (2020). Rhizoma Coptidis for alzheimer’s disease and vascular dementia: a literature review. *Current Vascular Pharmacology*.

[7] Zhu D. (2012). *Danxi’s Mastery of Medicine*.

[8] Park K. D., Lee J. H., Kim S. H., Kang T. H., Moon J. S., Kim S. U. (2006). Synthesis of 13-(substituted benzyl) berberine and berberrubine derivatives as antifungal agents. *Bioorganic & Medicinal Chemistry Letters*.

[9] Liu F., Zhang Z., Lai J., Hu B. (2010). Determination of four kinds of alkaloids from rhizoma coptis and processed rhizoma coptis by HPLC. *Chinese Traditional Patent Medicine*.

[10] Wang G. (1991). *Bo Ji Fang*.

[11] Chang M. (2015). *Notes on Rihuazi Medica*.

[12] Chinese Medical Science Press (2020). *Pharmacopoeia of the People’s Republic of China*.

[13] Sham T. T., Yuen A. C., Ng Y. F., Chan C. O., Mok D. K., Chan S. W. (2013). A review of the phytochemistry and pharmacological activities of raphani semen. *Evidence-Based Complementary and Alternative Medicine: ECAM*.

[14] Ghayur M. N., Gilani A. H. (2006). Radish seed extract mediates its cardiovascular inhibitory effects via muscarinic receptor activation. *Fundamental & Clinical Pharmacology*.

[15] Li Y. L., Jiang Y. H., Yang C. H., Sun J. C., Wang M. M., Yang W. Q. (2015). Enhanced protective effect of the combination of uncaria and semen raphani on vascular endothelium in spontaneously hypertensive rats. *Evidence-Based Complementary and Alternative Medicine: ECAM*.

[16] Gong Q. (2016). *Science of Processing Chinese Materia Medica*.

[17] Lv W., Ren T., Su Y., Meng X. (2011). Inhibition of glucoraphenin enzymolysis in Raphani Semen by processing. *Zhongguo Zhongyao Zazhi*.

[18] Sonmezdag A. S., Kelebek H., Selli S. (2016). Characterization of aroma-active and phenolic profiles of wild thyme (Thymus serpyllum) by GC-MS-Olfactometry and LC-ESI-MS/MS. *Journal of Food Science and Technology*.

[19] Gao S., Wang J., Qin W., Wang Y., Cheng L., Yang Y. (2021). Study on characteristic fingerprint of raw and processed raphani semen and changes of four chemical components. *Chinese Journal of Information on Traditional Chinese Medicine*.

[20] Lv W., Ren T., Su Y., Meng X. (2011). Inhibition of glucoraphenin enzymolysis in Raphani Semen by processing. *China Journal of Chinese Materia Medica*.

[21] Zhu L., Yu S., Zhang X., Zhou H., Sheng H. (2019). Analysis on changes of chemical components in enzymolysis process of raphani semen pieces based on HPLC-DAD characteristic spectra. *Chinese Journal of Experimental Traditional Medical Formulae*.

[22] Malik M. S., Riley M. B., Norsworthy J. K., Bridges W. (2010). Glucosinolate profile variation of growth stages of wild radish (Raphanus raphanistrum). *Journal of Agricultural and Food Chemistry*.

[23] Okunade O. A., Ghawi S. K., Methven L., Niranjan K. (2015). Thermal and pressure stability of myrosinase enzymes from black mustard (Brassica nigra L. W.D.J. Koch. var. nigra), brown mustard (Brassica juncea L. Czern. var. juncea) and yellow mustard (Sinapsis alba L. subsp. maire) seeds. *Food Chemistry*.

[24] Iori R., Barillari J., Gallienne E., Bilardo C., Tatibouët A., Rollin P. (2008). Thio-functionalised glucosinolates: unexpected transformation of desulfoglucoraphenin. *Tetrahedron Letters*.

